# Electrochemotherapy as a Trigger to Overcome Primary Resistance to Anti-PD-1 Treatment: A Case Report of Melanoma of the Scalp

**DOI:** 10.3389/fonc.2021.742666

**Published:** 2021-09-16

**Authors:** Davide Quaresmini, Alessandra Di Lauro, Livia Fucci, Sabino Strippoli, Ivana De Risi, Angela Monica Sciacovelli, Anna Albano, Gaetano Achille, Massimo Montepara, Sabino Russo, Gabriella Tassone, Michele Guida

**Affiliations:** ^1^Rare Tumors and Melanoma Unit, IRCCS Istituto Tumori Giovanni Paolo II, Bari, Italy; ^2^Otolaryngology Unit, IRCCS Istituto Tumori Giovanni Paolo II, Bari, Italy; ^3^Pathology Unit, IRCCS Istituto Tumori Giovanni Paolo II, Bari, Italy

**Keywords:** melanoma, electrochemotherapy, immunotherapy, resistance, case report

## Abstract

**Background:**

Immunotherapy with immune checkpoint inhibitors is one of the main therapies for advanced melanoma. Nevertheless, albeit remarkable, immunotherapy results are still unsatisfactory as more than half of patients progress, and resistance to treatment still has a dramatic impact on clinical outcomes. Local treatments such as radiotherapy or electrochemotherapy (ECT), in addition to local control with palliative intent, have been shown to release tumoral neoantigens that can stimulate a robust systemic antitumor immune response.

**Case Presentation:**

We report the case of a patient with multiple nodular melanoma lesions of the scalp initially treated with local ECT. Soon after the procedure, multiple new lesions appeared close to the treated ones, therefore the patient started a systemic treatment with the anti-PD-1 nivolumab. The lesions of the scalp did not respond to immunotherapy, presenting a loco-regional spreading. To control the bleeding and painful lesions, we performed a second ECT, while continuing systemic immunotherapy. The treated lesions responded to the second procedure, while the other lesions continued progressing in number and dimension. Unexpectedly, after 2 months from the second ECT, the patient presented a progressive shrinkage of both treated and untreated lesions until complete remission. Concomitantly, he developed immune-related adverse events including grade 4 thyroid toxicity, grade 2 vitiligo-like depigmentation and grade 2 pemphigoid. At present, after 18 months from the first ECT and 14 months from the starting of anti-PD-1 immunotherapy, the patient is in good clinical condition and complete remission of disease still persists.

**Conclusion:**

This case highlights the potential role of ECT in increasing tumor immunogenicity and consequently in inducing a powerful immune response overcoming primary resistance to checkpoint inhibitor immunotherapy.

## Introduction

Systemic therapy for metastatic melanoma (MM) has dramatically changed in the past decades. Specific BRAF/MEK inhibitors for BRAF-mutant MM induced response rates of 70-80%, with a progression-free survival (PFS) of 11-15 months and a median overall survival (OS) of more than 2 years (1). Furthermore, immunotherapy using monoclonal antibodies directed against the checkpoint molecules CTLA-4 (cytotoxic T lymphocyte antigen-4) and PD-1 (programmed death antigen 1) or its ligand PD-L-1 induced response rates of about 15% and 40%, respectively, regardless of the presence of BRAF mutations and with usually durable responses (2, 3). Despite these important results, over half of patients do not respond to modern immunotherapy or relapse after a temporary response to either targeted or immunological treatments. For wild-type BRAF melanomas, anti-PD-1 agents are the first choice of treatment, and for patients with primary resistance or progressing to anti-PD-1 the prognosis is very poor with a second/further line including the anti-CTLA-4 ipilimumab, cytotoxic chemotherapy, or drugs in experimental trials.

As known, tumor associated antigens (TAA) are neoantigens produced by tumor cells capable to elicit an immune response, mainly mediated by cytotoxic-T lymphocytes (4). Many attempts are being made to increase tumor immunogenicity and to induce a more powerful immune response including systemic or intratumoral co-adjuvant molecules, radiations, cytotoxic drugs (5, 6). Moreover, the abscopal effect has been reported in some patients, consisting in a response to a local therapy, most often radiation, not only at the treated lesion site but also systemically, leading to a shrinkage of untreated, distant lesions (7). Trials using concurrent or sequential radiotherapy and immunotherapy are ongoing with the aim of exploiting their synergistic effects and identifying the best timing for the two treatments (8). Electrochemotherapy (ECT) is a strategy associating the electroporation of tumor cells with the simultaneous administration of antineoplastic drugs, generally bleomycin. The phenomenon of reversible electroporation increases cell permeability when a pulsed electrical current is applied to tissues. This enhances the cytotoxicity of bleomycin with minimal systemic side effects. Small tumor size predicts better response, durable tumor control, and fewer side-effects (9, 10). It has been reported that in addition to direct cell death, ECT is able to induce a local and systemic immune response thanks to its capability to release TAA from electroporated tumor cells (11–13). Thus, it has been hypothesized that the combination of ECT with systemic immunotherapy could constitute an effective strategy to induce an immunological durable and strong response against the tumor (14–17). So far, apart from anecdotal cases reported in literature (18–21), there are no studies that have verified this association on a large patient population, nor has the possibility of reverting resistance to immunotherapy been explored with the use of local treatments such as ECT.

Here, we report the case of an elderly patient with a locally advanced melanoma of the scalp presenting primary resistance to the anti-PD-1 nivolumab that was reverted after two ECT procedures.

## Case Presentation

### Clinical History

**Figure 1:**
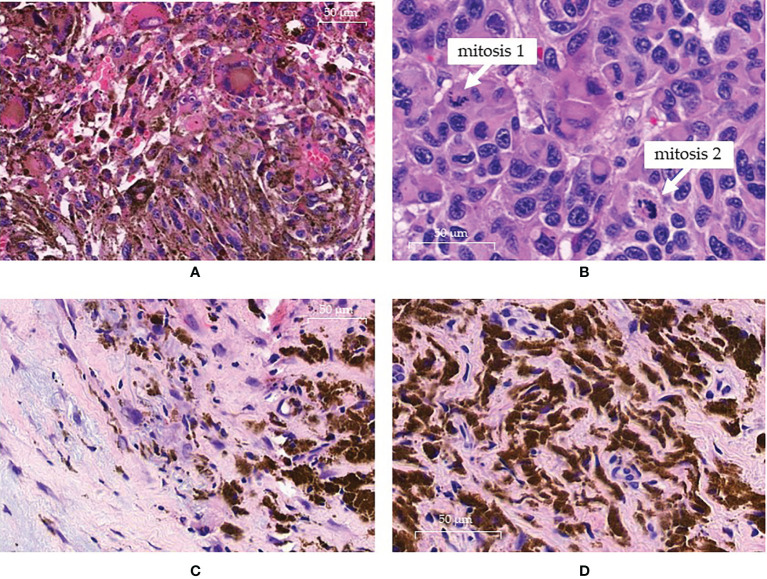
Pathology findings. **(A, B)** pre-treatment histology showing the presence of atypical neoplastic clones at high cellularity in the same sample: **(A)** field with melanocytic clone of cells rich in cytoplasmatic melanic pigment; **(B)** other field with an amelanotic clone displaying intense mitotic activity. **(C, D)** histologic sampling at complete response: **(C)** absence of neoplastic cells replaced by loose and dense fibrotic tissue with histiocytes; **(D)** histiocytes show cytoplasm intensely filled with melanic pigment.

**Figure 2:**
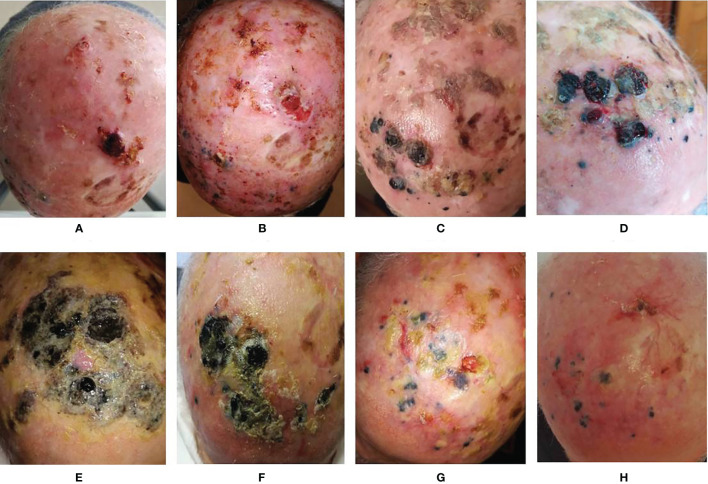
Evolution of the lesions. **(A)** Basal evaluation; **(B)** 2 weeks after the first ECT; **(C)** 2 months after the first ECT and 1 month after 1 cycle of anti-PD1; **(D)** 3 months after the first ECT and 1 month after 2 cycles of anti-PD1; **(E)** 1 week after the second ECT; **(F)** 2 months after second ECT and completion of 5 cycles of anti-PD1; **(G)** 3 months after second ECT and anti-PD1 temporary suspension for toxicity; **(H)** 17 months after the second ECT and after 15 cycles of anti-PD1.

In February 2020 the patient G.A. came to our Melanoma and Rare Tumors Unit for a relapsed, cutaneous melanoma of the scalp. The patient was an 85-years old caucasian male presenting with a type 2 phototype, a history of chronic sun exposure, positive familial history for melanoma to his sister, and a personal history of multiple cancers of the skin. He had undergone multiple resections for basal cell cutaneous carcinomas of the head and neck region. In 2011 he had been diagnosed a Merkel cell carcinoma of the right parietal region of the scalp treated with surgical resection and local adjuvant radiotherapy. In March 2013 he underwent the excision of a melanoma at left frontal area of the scalp. In October 2019, the patient noted the onset of a pigmented cutaneous nodulation of about 2 cm in diameter at the left parietal skin of the scalp which was surgically removed resulting in a relapse of malignant melanoma, 3.1 mm in thickness, non-ulcerated, with 11 mitoses/mm^2^ and negative surgical margins (**Figures 1A, B**). The BRAF status resulted wild-type. Soon after the excision, additional multiple (12 visible) millimetric pigmented satellite lesions appeared close to the surgical scar. The CT scan was negative for other locoregional or distant localizations, but the patient was judged surgically unfit because of the high number of lesions and the rapid evolution of the disease. In February 2020, the patient came to our Institution for the first time, and at the physical examination he presented diffused pigmented lesions of the left fronto-parietal area of the scalp 2 to 20 mm large, mostly ulcerated. The patient complained of frequent bleeding from the ulcerated lesions, continuous, severe pain at his scalp and was in serious concern for his life (**Figure 2A**). According to the American Joint Committee on Cancer VIII edition classification, the disease resulted a T3bN2cM0 (stage IIIC) cutaneous melanoma.

### Treatments and Responses

The case was discussed at our multidisciplinary melanoma tumor board concluding for a local treatment with electrochemotherapy (ECT) in order to obtain a rapid relief of symptoms and a good local control of disease. After obtaining informed consent, in February 2020 the patient was treated with ECT under general anesthesia at our Otolaryngology Unit according to the standard procedure (22). Briefly, bleomycin was administered intravenously as a bolus, at the dose of 15,000 IU/m^2^. After 8 minutes, short (8 pulses of 100 μs duration, at a repetition frequency of 5,000 Hz), high‐voltage (400–960 V) electric pulses were delivered to the tumoral lesions by means of a needle electrode. The electric pulses were generated by a square wave pulse generator suitable for clinical electroporation (Cliniporator™, IGEA, Carpi, Italy). The patient obtained a good response of all treated lesions. Nevertheless, after a few days, new lesions appeared in the proximity of the treated area (**Figure 2B**). Thus, in April 2020 a systemic immunotherapy with the anti-PD-1 nivolumab was started at the flat dose of 480 mg intravenously every 4 weeks. Unfortunately, independently of treatment, the lesions increased in dimension, extension and number with some of them becoming ulcerated and bleeding (**Figures 2C, D**). The CT scan performed after 3 cycles of therapy persisted negative for distant metastases. Thus, in order to locally control the disease, a second ECT procedure was performed in July 2020, continuing systemic therapy. As expected, the lesions treated with ECT started to reduce but other new lesions rapidly appeared (**Figure 2E**). Due to the good clinical conditions of the patient and to the limited therapeutic options available, we decided to continue the anti-PD-1 treatment. After 2 further cycles of nivolumab, we observed an initial slow shrinkage of all lesions (**Figure 2F**). At the same time, the patient presented a grade 4 hypothyroidism with marked weakness, hypersomnia, bradypsychism, and bradycardia. According to the current toxicity guidelines, the anti-PD-1 treatment was promptly suspended and hormonal substitutive therapy was started with a progressive normalization of thyroid function and disappearance of symptoms. Immunotherapy was then started again (**Figure 2G**) and is currently ongoing. Interestingly, a few weeks after the acute endocrine toxicity, multiple depigmented areas appeared on the skin, in a vitiligo-like phenotype involving more than 10% of the skin of the scalp and face, the trunk and the extremities. The patient continued to respond until the melanoma lesions became only flat, pigmented, non-ulcerated areas. A biopsy performed at this time showed the absence of vital neoplastic cells, with wide areas of histiocytes filled with melanotic pigment, associated to fibrosis in correspondence to the ECT-treated areas (**Figures 1C, D**). Therefore, further immunohistochemical investigations were not feasible. After 15 cycles of Nivolumab, the patient presented a diffuse blistering and bullous rash involving the skin at his trunk and inferior limbs. Many lesions were eroded and infected with purulent secretion. Given the clinical suspect of autoimmune bullous dermatosis, autoantibodies were dosed and showed anti-BP180 positivity (116 relative units/mL; normal cut-off value < 20). Finally, a biopsy performed at one bullous lesions of the lower limbs showed the presence of a sub-epidermic bulla with modest lymphoplasmacytic infiltration prevailing at the perivascular dermal areas. Such data led to the diagnosis of pemphigoid for which corticosteroid therapy was administered in association to systemic antibiotics to treat the bacterial superinfection. The immunotherapy was interrupted, as the patient is still recovering and corticosteroid therapy is being tapered before suspension at the time of writing this report.

**Figure 3:**
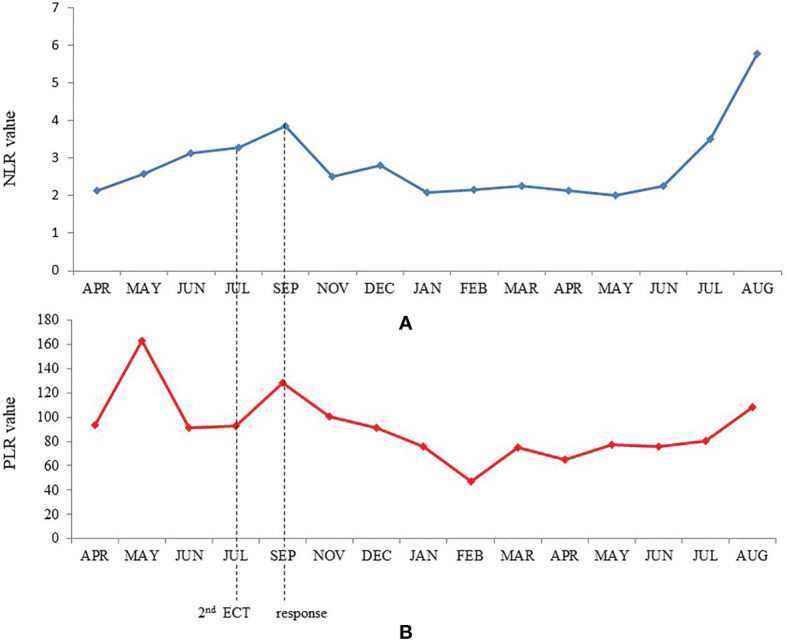
Trends of neutrophil-lymphocyte ratio **(A)** and platelet-lymphocyte ratio **(B)** during treatment.

**Figure 4:**
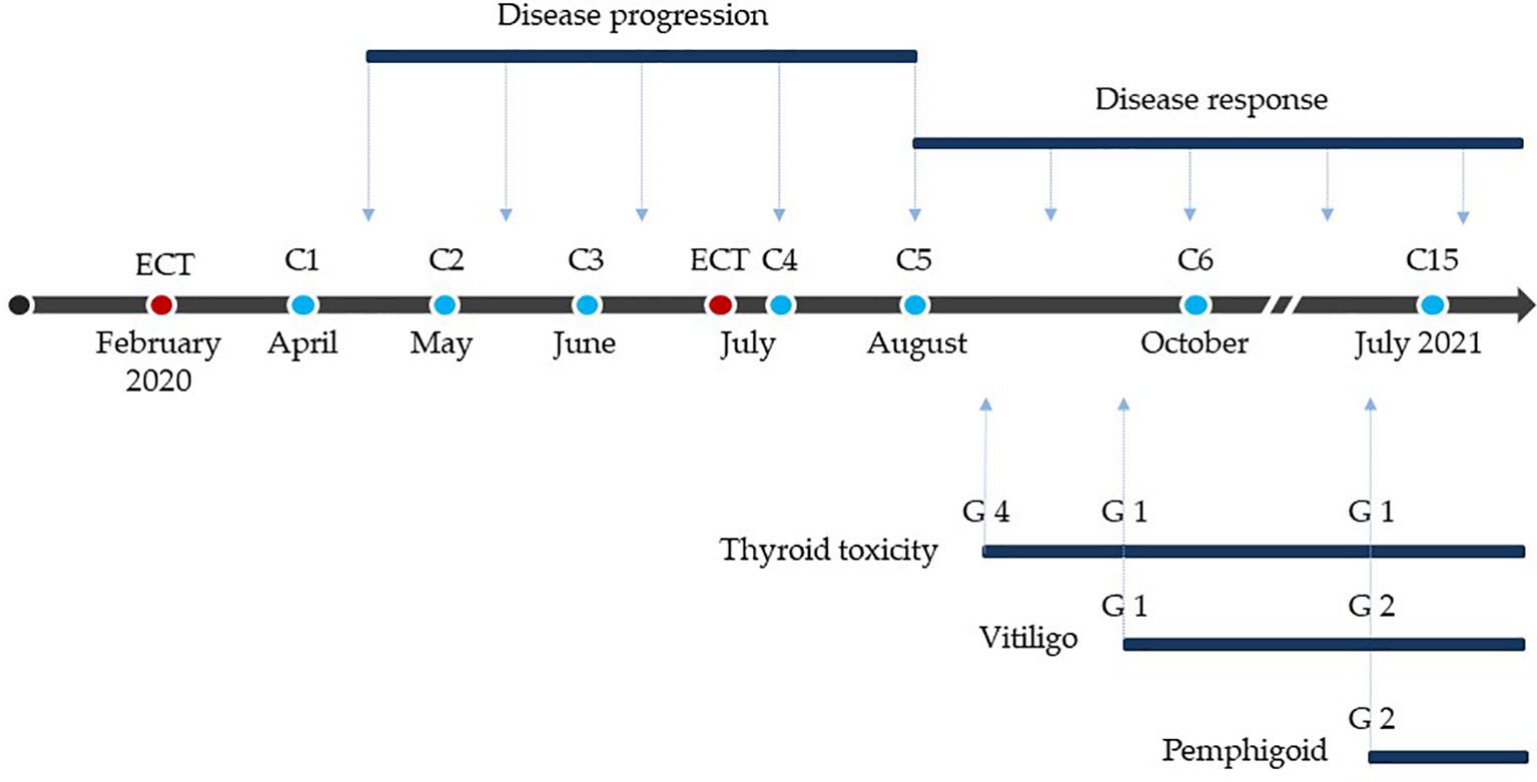
Treatment, response and toxicity. Red dots: electrochemotherapy. Blue dots: anti-PD-1 treatments. C: cycle of anti-PD-1. G: grade of toxicity according to Common Terminology Criteria for Adverse Events (CTCAE) classification, 5.0 version.

We also analyzed peripheral blood parameters [total leukocyte count, neutrophils, lymphocytes, monocytes, platelets, neutrophils/lymphocytes ratio (NLR), platelets/lymphocytes ratio (PLR)] performed before the first ECT and then every month since the start of immunotherapy to compare their behaviors with clinical outcomes. Of note, leukocytes and neutrophils slightly increased while platelets presented an initial peak followed by a progressive return to basal values until the onset of a clinical response. Subsequently, only platelets presented a further reduction, yet within the normal range values. Therefore, NLR and PLR progressively increased until clinical response, then they both fell to a minimum value while maintaining a constant plateau level. NLR, and at a minor degree also PLR, presented an increase in their value over the last 2 months of treatment, concomitantly with the appearance of the superinfected pemphigoid lesions of the skin (**Figure 3**). At present, after 18 months from the first ECT and after 14 months from the beginning of anti-PD-1 immunotherapy, the patient is in good clinical condition with complete remission of disease (**Figure 2H**). The anti-PD-1 immunotherapy was recently interrupted due to the grade 2 pemphigoid adverse reaction, and the clinical condition of the patient is improving with the still ongoing corticosteroid therapy. Overall, the patient is very satisfied with the results of treatment. He was deeply aware of the poor prognosis associated with his condition before immunotherapy and the prompt recognition and management of the adverse events together with the high compliance of the patient himself allowed to handle the side effects and minimize their impact on the overall quality of life. The clinical case is visually summarized in **Figure 4**.

## Discussion

We report the case of an elderly patient with melanoma of the scalp relapsed after surgical resection and then successfully treated with ECT and systemic immunotherapy. The patient showed a primary resistance to the anti-PD-1 nivolumab that was reverted after the second ECT procedure. ECT is an established local ablative treatment for primary or metastatic tumoral lesions with different histologies able to induce a response rate of 80% regardless of the histological type, particularly in superficial lesions that are more easily approached with this technique (9, 10, 22). Moreover, in selected patients with metastatic disease, ECT represents a useful tool to control oligoprogression allowing the continuation of systemic treatment and prolongation of overall survival (23). ECT has also been shown to induce a local and systemic immune response. ECT followed by CpG oligodeoxynucleotide injection is able to trigger both potent local synergistic antitumor effects and a systemic antitumor response at untreated tumor lesions (11). Moreover, ECT induces a massive recruitment of macrophages and CD3+ CD8+ T cells with a decrease of CD4+ FOXP3+ T regulatory cells in melanoma metastases (12). Finally, ECT promotes Langerhans cells migration from the tumor to the draining lymph nodes and plasmacytoid and dermal dendritic cells recruitment at the site of the lesion (13).

In our patient, a response to systemic immunotherapy was noted after the second ECT procedure. We hypothesized that the response of the patient’s disease was induced by the release of TAA and their presentation by the antigen presenting cells to T-lymphocytes, turning a non-T cell inflamed into T-cell inflamed tumor with consequent strong antitumoral effects. Unfortunately, the complete absence of cancer cells observed at the second biopsy of the lesions did not allow to perform any further immunohistochemical and molecular investigations to confirm our hypothesis. Interestingly, after 6 cycles of anti-PD1 treatment, the patient developed a vitiligo-like skin depigmentation that is the known expression of CD8^+^ activation against antigens shared by melanoma and melanocytes, and has been strongly associated with better outcomes in melanoma patients treated with immunotherapy (24). Moreover, the onset of grade 4 hypothyroidism at the initial response also demonstrated the synergistic antineoplastic activity induced by ECT and anti-PD-1. Also the pemphigoid that the patient recently developed is an immune-mediated condition, and its onset is to be attributed to the anti-PD-1 treatment. Of interest, at the analysis of peripheral blood parameters we found a progressive increase of the neutrophils/lymphocytes ratio (NLR) and platelets/lymphocytes ratio (PLR) during the non-response phase; then, at the initial tumor shrinkage, both of these parameters fell to a minimum value while maintaining a constant plateau level. It has been recently reported that NLR and PLR are prognostic biomarkers in different types of cancers including melanoma (25). These findings derive from the demonstration that neutrophils and platelets contribute to cancer cell growth and migration and from the evidence of the inter-relationship between thrombosis, inflammation and cancer immune surveillance (26). Moreover, they seem predictive of a worse efficacy of both chemotherapy and immunotherapy (27, 28). Finally, an early increase of NLR during treatment with PD-1 inhibitor monotherapy seems able to identify patients with a shorter survival and correlate with time to treatment failure (27). In our patient, NLR and PLR presented an increase over the latest months of treatment while maintaining complete response, as a consequence of a neutrophilic leukocytosis which is referrable to the purulent superinfection of the pemphigoid lesions that he developed.

In our case, ECT changed the quality of life of our patient, but it also actually changed his response to checkpoint inhibitor immunotherapy with a positive impact on his PFS and OS. Thus, it could represent a potential innovative potential innovative strategy in combination with systemic immunotherapy in order to generate a strong systemic long-lasting antitumoral immunity against melanoma. These results might contribute to design novel trials to verify this combination in a large patients’ population.

## Data Availability Statement

The raw data supporting the conclusions of this article will be made available by the authors, without undue reservation.

## Ethics Statement

Ethical review and approval was not required for the study on human participants in accordance with the local legislation and institutional requirements. The patients/participants provided their written informed consent to participate in this study. Written informed consent was obtained from the individual(s) for the publication of any potentially identifiable images or data included in this article.

## Author Contributions

Conceptualization, MG, SS, and DQ. Data collection, IR, DQ, SS, and MG. Methodology, DQ, LF, and AL. Analysis, writing, and editing, DQ, AL, LF, SS, IR, AS, AA, GA, MM, SS, SR, GT, and MG. Supervision, MG and DQ. All authors contributed to the article and approved the submitted version.

## Conflict of Interest

The authors declare that the research was conducted in the absence of any commercial or financial relationships that could be construed as a potential conflict of interest.

## Publisher’s Note

All claims expressed in this article are solely those of the authors and do not necessarily represent those of their affiliated organizations, or those of the publisher, the editors and the reviewers. Any product that may be evaluated in this article, or claim that may be made by its manufacturer, is not guaranteed or endorsed by the publisher.
